# Relation between abnormal synergy and gait in patients after stroke

**DOI:** 10.1186/1743-0003-11-141

**Published:** 2014-09-25

**Authors:** Kaoru Sakuma, Koji Ohata, Keisuke Izumi, Yu Shiotsuka, Tadashi Yasui, Satoko Ibuki, Noriaki Ichihashi

**Affiliations:** Department of Physical Therapy, Human Health Sciences, Graduate School of Medicine, Kyoto University, 53, Kawahara-cho, Kyoto Sakyo-ku, 606-8507 Japan; Japan Society for the Promotion of Science, 5-3-1 Kojimachi, Tokyo, 102-0083 Japan; Department of Rehabilitation, Biwako Gakuen Medical and Welfare Center, 8-3-113 Kasayama, Shiga, 525-0072 Japan; Department of Rehabilitation, Hakuhoukai Tagawashinsei Hospital, Fukuoka Tagawa-shi, 825-0004 Japan; Manufacturing Technology Section, Kawamura Gishi Co., Ltd, 1-12-1 Goryo, Daito-shi, Osaka, 574-0064 Japan

**Keywords:** Abnormal synergy, Gait, Stroke

## Abstract

**Background:**

The abnormal synergy seen in patients after stroke is considered to limit the ability of these patients. However, in the lower extremity, antigravity torque generation rather than precise movement is needed for functions such as sit-to-stand movement and gait. Therefore, the ability to generate torque may be important either as a primary movement or as an abnormal synergy. We attempted to quantify the torque generation in the lower limb, selectively and as an abnormal synergy, and its relation with gait.

**Methods:**

Selectively generated plantar flexion torque in the ankle and plantar flexion torque secondarily generated accompanying maximal hip extension (i.e., torque generated with abnormal synergy) were measured in subjects after stroke and control subjects. In subjects after stroke, secondary torque generation while controlling hip extension torque as 25%, 50%, and 75% of the maximal hip extension was also measured. The relation of torque generation with the gait speed and timed-up-and go test (TUG) was also analyzed.

**Results:**

In subjects after stroke, there was no difference between the amount of plantar flexion torque generated secondarily and the selectively generated torque, whereas the selective torque was significantly greater in control subjects. Pearson product–moment correlation coefficient analysis revealed that TUG speed is related to secondarily generated torque accompanying maximal hip extension but not with selectively generated torque.

**Conclusion:**

Secondarily generated torque was found to be a factor that affects TUG speed, and the ability to generate torque even through abnormal synergy may help for gait ability in subjects after stroke.

**Electronic supplementary material:**

The online version of this article (doi:10.1186/1743-0003-11-141) contains supplementary material, which is available to authorized users.

## Background

After stroke, many patients experience motor impairments [1] that cause gait deficits [2–7]. The ability to perform activities of daily living depends on gait ability [2]. Therefore, it is important to understand the relation between impairment and gait deficit in patients after stroke.

Abnormal synergy is a motor impairment in patients after stroke [8, 9]. Some patients lose independent control of selected muscle groups, resulting in coupled joint movements that are often inappropriate for the desired task. These coupled movements are known as abnormal synergy. For the lower limb, abnormal synergy is grouped into extension synergy (internal rotation, adduction, and extension of the hip; extension of the knee; and extension and inversion of the ankle) and flexion synergy (external rotation, abduction, and flexion of the hip; flexion of the knee; and flexion and eversion of the ankle) [9]. Abnormal synergy has been recognized as a factor limiting the motor rehabilitation of patients after stroke [10–14]. Studies measuring the relative phase in stroke patients have shown that patients after stroke differ from normal subjects in intra- [15–17] and inter-limb [18] coordination of the lower limb. Difference in kinematics is also seen in stroke subjects [19]. Many patients recover gait activity with compensatory adaptation [19–22]. A previous study showed that the Fugl-Meyer synergy score was significantly correlated with gait speed [23]. It was also found that attempts to make gait patterns resemble those of neurologically healthy adults by using Lokomat or a robotic knee orthosis did not result in the extinction of abnormal synergy [24, 25]. The ability to exert lower-limb muscle torque is important for gait [3, 6, 26, 27]. By quantitatively assessing the abnormal synergy in the lower limb by measuring the torque exerted as abnormal synergy, and then investigating its relation with gait ability, we may be able to gain insight into the relation between abnormal synergy and gait ability. In a study quantifying the upper-limb abnormal synergy in patients after stroke, Dewald et al. [28, 29] measured the joint torque that the subjects were attempting to maximize as the primary torque and the torques at other directions as secondary torques, along with electromyographic (EMG) measurements during voluntary isometric muscle contractions. They found that patients after stroke had significantly decreased maximum voluntary torque compared with the control subjects, and that the patients exhibited reductions in maximum torques when required to control the secondary torque [28, 29]. Patients after stroke could not generate voluntary torque selectively without generating torque in other joints. Other studies that applied a similar method to the lower limb [13, 14] suggested that the primary contributor to lower-limb motor deficits was the weakness of voluntary torque rather than the value of abnormal synergy.

Patients after stroke lack the ability to selectively generate voluntary torque without generating joint torque in other directions and in other joints both in the upper and lower limbs. We hypothesized that abnormal synergy in the lower limb may compensate for the weakness of agonist muscle in patients after stroke. Furthermore, we hypothesized that if the previous hypothesis is correct, a relation between abnormal synergy of the lower limb and gait would also be observed. However, the relation between of the lower limb and gait has not yet been clarified.

To study the abnormal synergy of the lower limb and its relation with gait, we measured the ankle joint torque generated secondarily during maximum voluntary isometric hip extension (secondary torque, STo) and the maximum voluntary ankle plantar flexion torque primarily generated without hip extension torque generation (primary torque, PTo). Then, we investigated whether PTo and STo are related to the gait speed or TUG in patients after stroke.

## Methods

### Subjects

Eleven subjects after stroke and 13 control subjects participated in this study. The characteristics of the subjects are summarized in Table 1.

**Table 1:** **Characteristics of the subjects**

	Subjects after stroke (n = 11)	Control subjects (n = 13)
Age (years)	51.1 ± 10.7	51.1 ± 9.6
Sex (n): M/F	8/3	13/0
Height (cm)	164.5 ± 7.4	169.0 ± 6.3
Weight (kg)	60.3 ± 8.7	67.6 ± 10.9
B.R.S. (n): III/IV/V/VI	2/4/3/2	
Time since onset (months)	90.7 ± 79.9	
Paretic side (n): R/L	7/4	

F, female; M, male.

R, right; L, left.

Values are presented as means ± standard deviation (SD), unless otherwise indicated.

The subjects were recruited in the community through convenience sampling. The subjects after stroke satisfied the following criteria: (i) hemiparesis resulting from a unilateral cortical or sub-cortical brain lesion; (ii) >6 months since onset; and (iii) able to walk in the community. The exclusion criteria for the subjects after stroke were as follows: (i) history of other neurologic, respiratory, cardiovascular, or orthopedic problems that influence their participation in the study; (ii) brainstem or cerebellar lesions; and (iii) inability to provide informed consent. Control subjects with similar age to the subjects after stroke were recruited. Control subjects were excluded if they had neurologic, respiratory, cardiovascular, or orthopedic problems. All subjects provided informed consent before participation in this study. This study was approved by the institutional review board of Kyoto University.

### Primary torque and secondary torque

**Figure 1:**
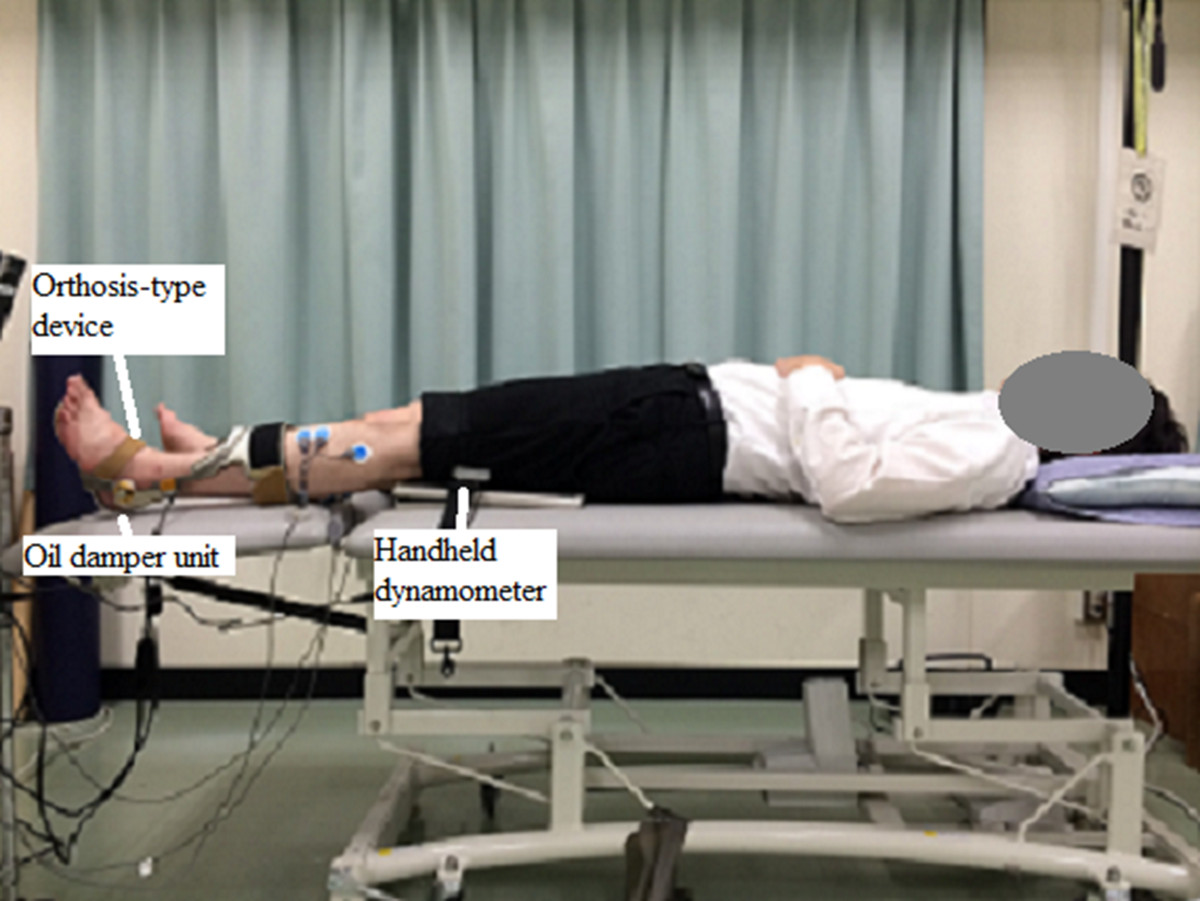
**Schematic of the test setup.** The subject was placed in the supine position. The subject’s foot was attached to the orthosis-type device with a load cell, and the thigh was attached to the handheld dynamometer. Upon the generation of the plantar flexion torque by the subject, the hydraulic cylinder pushed the load cell as a counterforce. The counterforce, which reflects the plantar flexion torque, was numerically indicated in a personal computer linked to the device.

A schematic of the test set-up is shown in Figure 1. Measurements were taken for all subjects in the supine position (hip extension angle of 0°, knee extension angle of 0°, and ankle dorsiflexion angle of 0°). The measured side was the paretic lower limb for subjects after stroke and the right limb for control subjects.

For recording the PTo and STo, an originally developed orthosis-type device with a load cell that could measure the ankle plantar flexion torque (Gait Solution Design; Kawamura Gishi, Osaka, Japan [30]) was used. A load cell was inserted above the hydraulic cylinder in the oil damper unit. Upon the generation of the plantar flexion torque by the subject, the hydraulic cylinder pushed the load cell as a counterforce; the counterforce, which reflects the planter flexion torque, was numerically indicated in a personal computer linked the device [30]. For recording the hip extension torque, a handheld dynamometer (μ-tas F-1; ANIMA Corp., Tokyo, Japan) was used. The subject’s foot was attached to the orthosis-type device and the thigh was attached to the handheld dynamometer. The PTo and the STo during maximum voluntary hip extension torque were each measured once in both the control subjects and the subjects after stroke. The subjects started in a relaxed state and slowly increased the torque generation to the maximum level, which was held for approximately 3 s.

In the subjects after stroke, secondary torque generation while controlling hip extension torque as 25%, 50%, and 75% of the maximal hip extension (25%STo, 50%STo, and 75%STo, respectively) was also measured in a random order. The subjects were provided with visual feedback about their exerted hip extension torque, measured using the handheld dynamometer, to aid them in exerting a force matching the target torque. The subjects practiced two to three times until they could control their hip extension torque at the target value. The ankle plantar flexion torque was measured once for each target torque for 3 s where the generated hip extension torque matched the target torque.

The plantar flexion torque collected in each trial was computerized concurrently with the EMG data. The ensemble ankle plantar flexion torque in the 500-ms window was normalized to body weight (Nm/kg).

The EMG recordings were collected by using the Telemyo 2400 T hardware (Noraxon USA Inc., Scottsdale, Arizona, USA; sampled at 1500 Hz, band-pass filter at 10-500 Hz). Bipolar surface electrodes (Blue Sensor; Medicotest Inc., Olstykke, Denmark) with an interelectrode distance of 20 mm (center-to-center) were placed on the muscle belly of the tibialis anterior, gastrocnemius (on the lateral head), and soleus muscles. Electrode placement on the tibialis anterior was at one-third on the line 1–2 cm lateral to the tibia; the gastrocnemius, at one-third on the line between the head of the fibula and the heel; and the soleus, at one-half to two-third on the line between the head of the medial condyles of the femur and the tip of the medial malleolus. A ground electrode was placed on the head of fibula. We prepared the electrode locations by cleaning the sites with alcohol wipes. The EMG data were smoothed by using a root-mean-square algorithm over a 10-ms window, and the mean value for 1-s duration was taken concurrently with the maximum ankle plantar flexion torque generation.

### Gait

The 10-m gait speed and TUG were measured in the subjects after stroke. In the 10-m gait test and TUG, the subjects wore an ankle foot orthosis that allowed ankle movement; however, they did not use a cane or any other walking aids. For recording the gait speed, the subjects after stroke were asked to perform gait trials twice at their preferred speed. The gait speed (m/s) was calculated from the mean of the 10-m gait time data. For recording the TUG, the time taken to stand up from a seated position, walk forward for 3 m, walk back, and then return to the seated position was measured once.

### Data analysis

One of the characteristics of abnormal synergy recognized as the limiting factors for the motor rehabilitation was that abnormal synergy becomes more prominent when the subjects attempt to generate higher joint torques [8, 9]. To assess whether these characteristics is reflected in the 25%STo, 50%STo, 75%STo, and STo, repeated-measures analysis of variance was used to analyze the effect of the exertion condition of hip extension on the ankle plantar flexion torque. Tukey’s test was used to examine the subsequent planned comparisons between each exertion condition.

Second, two-factor analyses of variance and t-test were used to analyze the difference between the group factor (subjects after stroke and control) and the exertion condition factor (PTo and STo) in the plantar flexion torque. The t test was used to analyze the difference between PTo and STo on EMG.

Finally, Pearson product–moment correlation coefficient was used to assess the relation between the gait speed or TUG and PTo or STo. Then, stepwise multiple linear regression analysis was used to assess the influence of PTo and STo on gait speed or TUG.

A significance level of 0.05 was used for all comparisons.

## Results

### Relation between secondary torque generation and percent hip extension torque in the subjects after stroke

**Figure 2:**
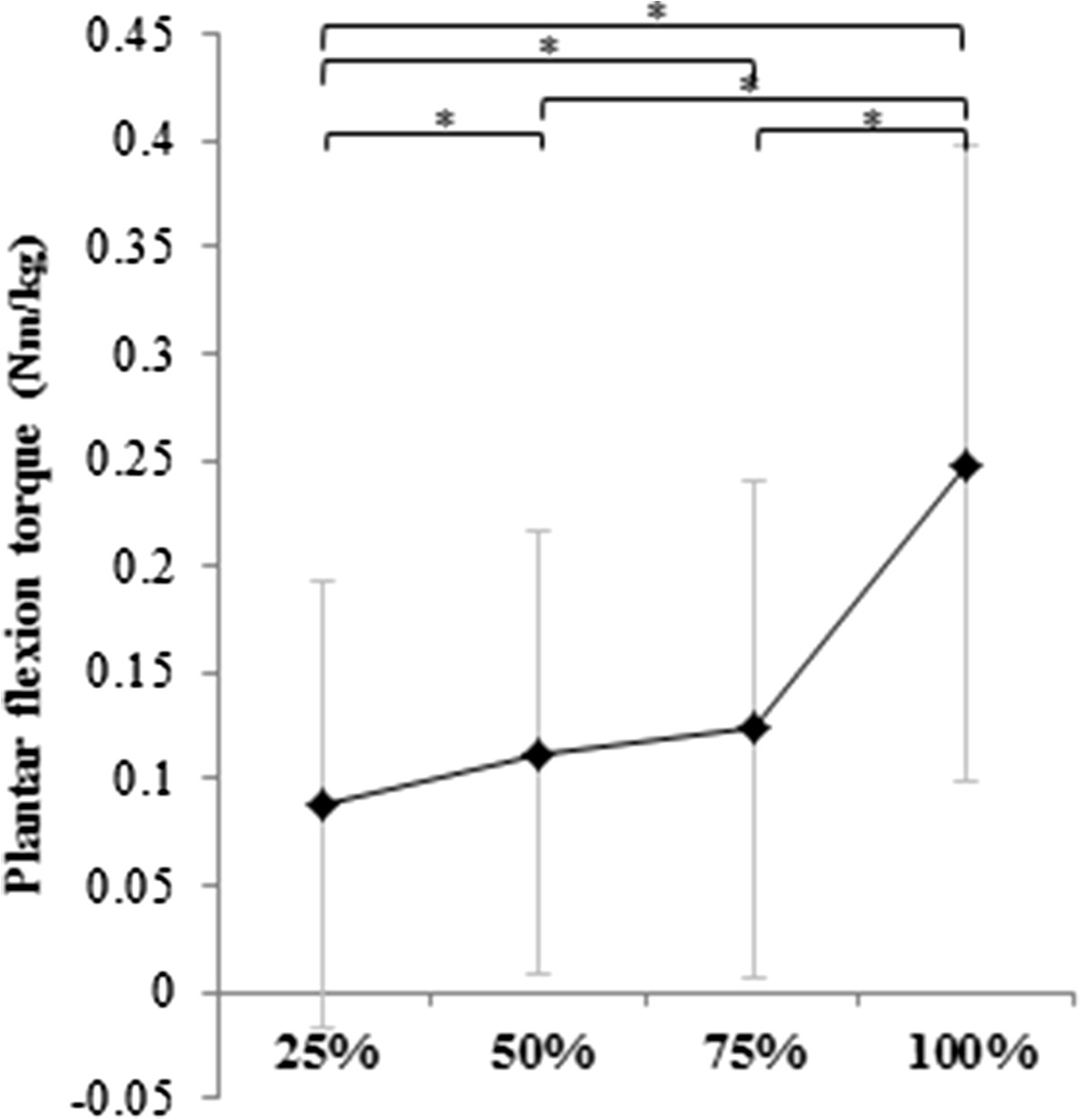
**Correspondence of secondary plantar flexion torque with percent hip extension torque in subjects after stroke.** An asterisk (*) denotes statistical significance between conditions (p < 0.05). The figure indicates the plantar flexion torque (standardized by body weight) generated when exerting the hip extension torque at 25%, 50%, 75%, and 100% of the maximal hip extension. There was a significant main effect of exertion condition. The secondarily generated plantar flexion torque (STo) corresponding to 50% of the maximum voluntary hip extension torque (50%STo) was higher than the 25%STo, and the 100% (STo) was higher than the 75%STo, 50%STo, and 25%STo. The 75%STo was higher than the 25%STo.

The correspondence of secondary plantar flexion torque with percent hip extension torque is summarized in Figure 2. There was a significant main effect of exertion condition (p < 0.01). The 50%STo was higher than the 25%STo, and the STo was higher than the 75%STo.

### Characteristics of primary torque and secondary torque in the subjects after stroke and the control group

**Figure 3:**
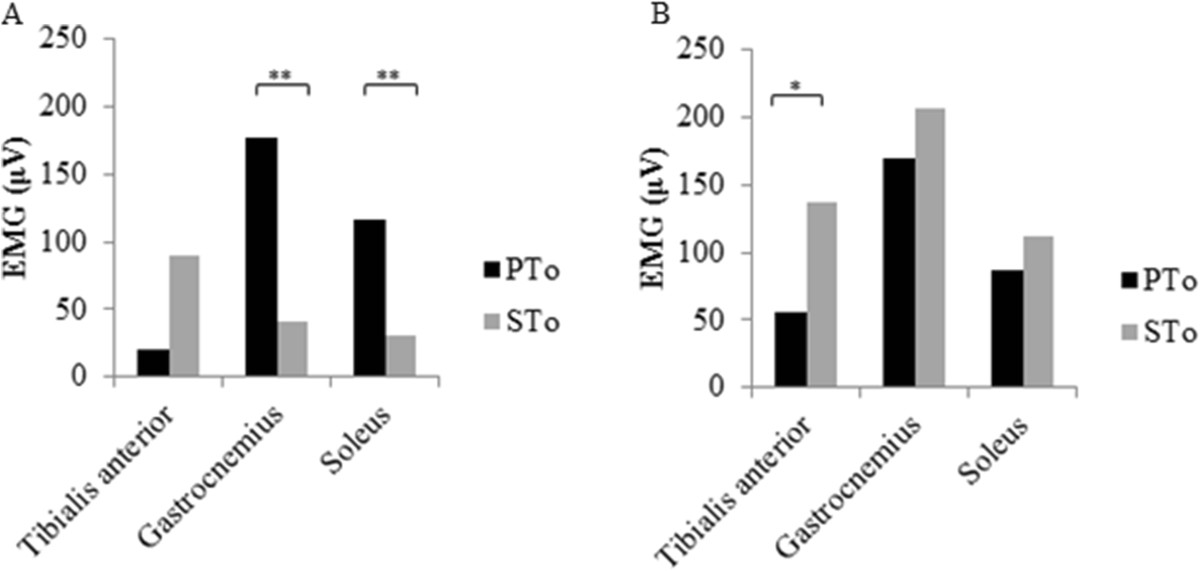
**Electromyography activity during primary torque and secondary torque generation.** An asterisk denotes statistical significance between conditions (**; p < 0.01, *; p < 0.05). **A**, In the control group, the gastrocnemius and soleus activities during PTo were higher than those during STo. **B**, In subjects after stroke, the tibialis anterior activity during STo was higher than that during PTo.

Interaction effects were found between the group factor and the exertion condition factor (p < 0.01). The subjects after stroke (0.1 ± 0.1) showed lower values than the controls in the PTo (0.6 ± 0.4, p < 0.05), but not in the STo (subjects after stroke, 0.2 ± 0.2; controls, 0.2 ± 0.2). The PTo was significantly higher than the STo in the control group (p < 0.01) but not in the subjects after stroke.The EMG data during PTo and STo are summarized in Figure 3. The gastrocnemius and soleus activities during PTo were higher than those during STo in the control group but not in the subjects after stroke. The tibialis anterior activity during the STo was higher than during PTo in the subjects after stroke but not in the control group.

### Gait

There were no relation between gait speed and the PTo (r = 0.4) or the STo (r = 0.5). There was a significant relation between TUG and the STo (r = 0.7) but not between TUG and the PTo (r = 0.5). Stepwise analysis revealed that the STo was a determinant of TUG (R^2^ = 0.5).

## Discussion

The results of this study revealed that in subjects after stroke, the increase in the percentage of maximum hip extension torque generated by the subjects increased the%STo. The subjects after stroke exhibited lower torque than the control group in the PTo but not in the STo. The PTo was significantly higher than the STo in the control group but not in the subjects after stroke. Our hypothesis was supported by the observation that there is a correlation between TUG and the STo in the subjects after stroke, and the principal finding of this study is that the STo was the determinant of TUG, as revealed by stepwise analysis. This is the first study that quantitatively demonstrated the inability to selectively generate voluntary torque in the lower limb, and showed that STo affects TUG in patients after stroke.

### Relation between secondary torque generation and percent hip extension torque in the subjects after stroke

Controlling agonist activity is a fundamental function required in activities of daily living. Therefore, in this study, we chose a task that requires generating a certain percentage of maximum voluntary torque. The subjects after stroke showed explicit increase in the %STo as the percentage of required hip extension increased, which is consistent with the characteristics of abnormal synergy described in previous studies [8, 9]. Therefore, the STo measured in this study is considered to reflect the feature of abnormal synergy.

### Characteristics of primary torque and secondary torque in the subjects after stroke and the control group

In a previous study that measured the secondary torque in the ankle joint during maximum voluntary hip extension in both controls and subjects after stroke, the secondary torque was seen in both groups and no differences were found in the rate of the secondary torque to maximum voluntary ankle plantar flexion torque between the groups [13]. Similarly in this study, there were concurrent ankle plantar flexion torques measured as the STo during the generation of the maximum voluntary hip extension torque in both the controls and the subjects after stroke, and there were no differences between the controls and subjects after stroke in the STo torque normalized to body weight. In the current study, the control group could generate considerably higher PTo than STo, whereas the subjects after stroke could only generate PTo torque equivalent to their STo torque. Moreover, the gastrocnemius and the soleus, which are the agonist muscles in ankle plantar flexion, were more activated during STo than during PTo in the subjects after stroke; the opposite was observed in the controls. This is probably due to the disorganization of motor unit recruitment, rate modulation patterns [31, 32], antagonist muscle weakness [33], and the abnormal corticospinal responses [34–36] that might affect the contribution of agonist activity to the voluntary torque seen in the subjects after stroke. It should also be noted that the tibialis anterior muscle activity during STo was higher than that during PTo in the subjects after stroke. The co-activation of the antagonist muscle, i.e., the tibialis anterior muscle might have inhibited the generation of plantar flexion torque as STo. However, the gastrocnemius and soleus activities during STo were not higher than those during PTo. Therefore, we conclude that the tibialis anterior muscle activity did not affect the results of this study. In the subjects after stroke, because of the inability to selectively activate the agonist muscle, the STo becomes relatively higher than the PTo.

### Correlation with gait ability

The stepwise analysis revealed that the STo, and not the PTo, was the determinant of TUG. Although there was no significant relation between the STo and gait speed, the correlation coefficient was high. Therefore, we consider that there might be a relation between the STo and gait ability. In previous studies, the relation between the Brunnstrom recovery stage [19] or the Fugl-Meyer assessment [23] and gait speed was reported. However, because these clinical assessments evaluated both recovery from abnormal synergy and improvement of voluntary movement, it was unclear whether abnormal synergy or voluntary movement was related to gait speed. In this study, by quantifying the abnormal synergy measuring the joint torque, the relation between abnormal synergy and gait was revealed for the first time, showing that STo was the determinant of TUG. On the other hand, a previous study showed that the paretic ankle plantar flexion torque was correlated with gait speed in the patients after stroke [27]. We consider that because TUG consists not only of gait but also of sit-to-stand movement, which requires a large torque [37, 38], abnormal synergy might be a related factor. Our results suggest that in subjects after stroke, STo might be adopted to compensate for the inability to generate the voluntary torque during gait.

### Limitation

Three limitations of this study need to be mentioned. First, the PTo and STo measured in this study might be affected by other factors such as co-activation and spasticity. Nevertheless, we could at least assess one aspect of abnormal synergy quantitatively as the joint torque generated concurrently with the intended voluntary torque.

Second, the subjects after stroke recruited in our study were community-dwelling, able to walk. Therefore, our results may not be applicable to patients in a more severe condition after stroke who are unable to live in the community or have greater disability in gait.

Finally, this study did not evaluate abnormal synergy during gait, or the relation of abnormal synergy with each element in gait. Therefore, the influence of abnormal synergy on each factor in gait remains unknown.

## Conclusion

We found that the amount of secondarily generated plantar flexion torque (STo) was as large as the selectively generated plantar flexion torque (PTo), and that the STo was negatively correlated to TUG speed. This suggests that torque generation as abnormal synergy may help for patients after stroke who cannot sufficiently generate selective torque.

## Authors’ original submitted files for images

Below are the links to the authors’ original submitted files for images. Authors’ original file for figure 1 Authors’ original file for figure 2 Authors’ original file for figure 3

## Abbreviations

PTo: The primary torque
STo: The secondary torque
TUG: Timed-up-and go test
EMG: Electromyographic.
