# Supplementary figures and images for: Relation between abnormal synergy and gait in patients after stroke
Source: J Neuroeng Rehabil. 2014 Sep 25;11:141. doi: 10.1186/1743-0003-11-141 (PMC4189205; doi:10.1186/1743-0003-11-141)

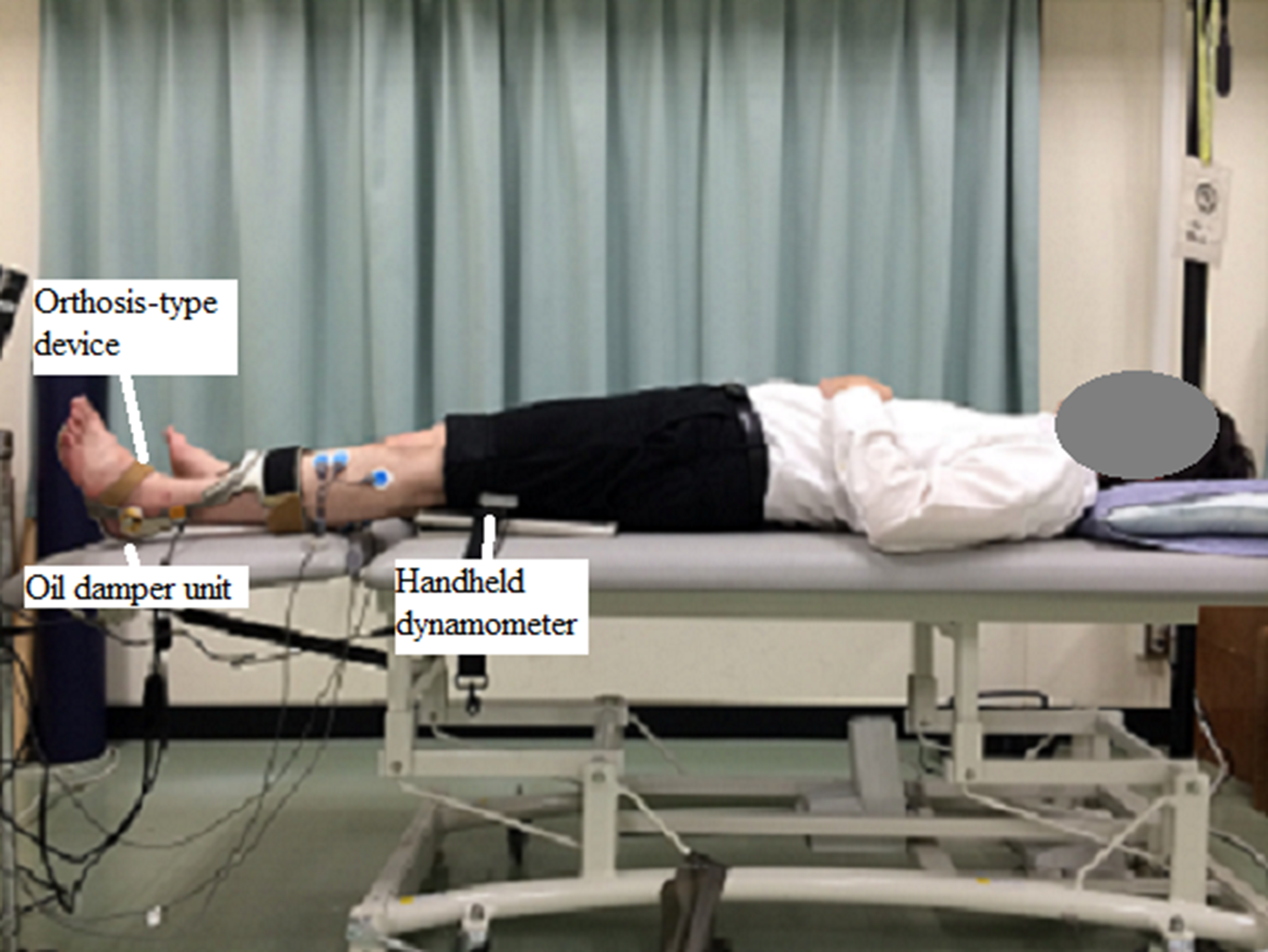

Supplement: Supplementary file 1 — Authors’ original file for figure 1 [file 12984_2014_661_MOESM1_ESM.tif]

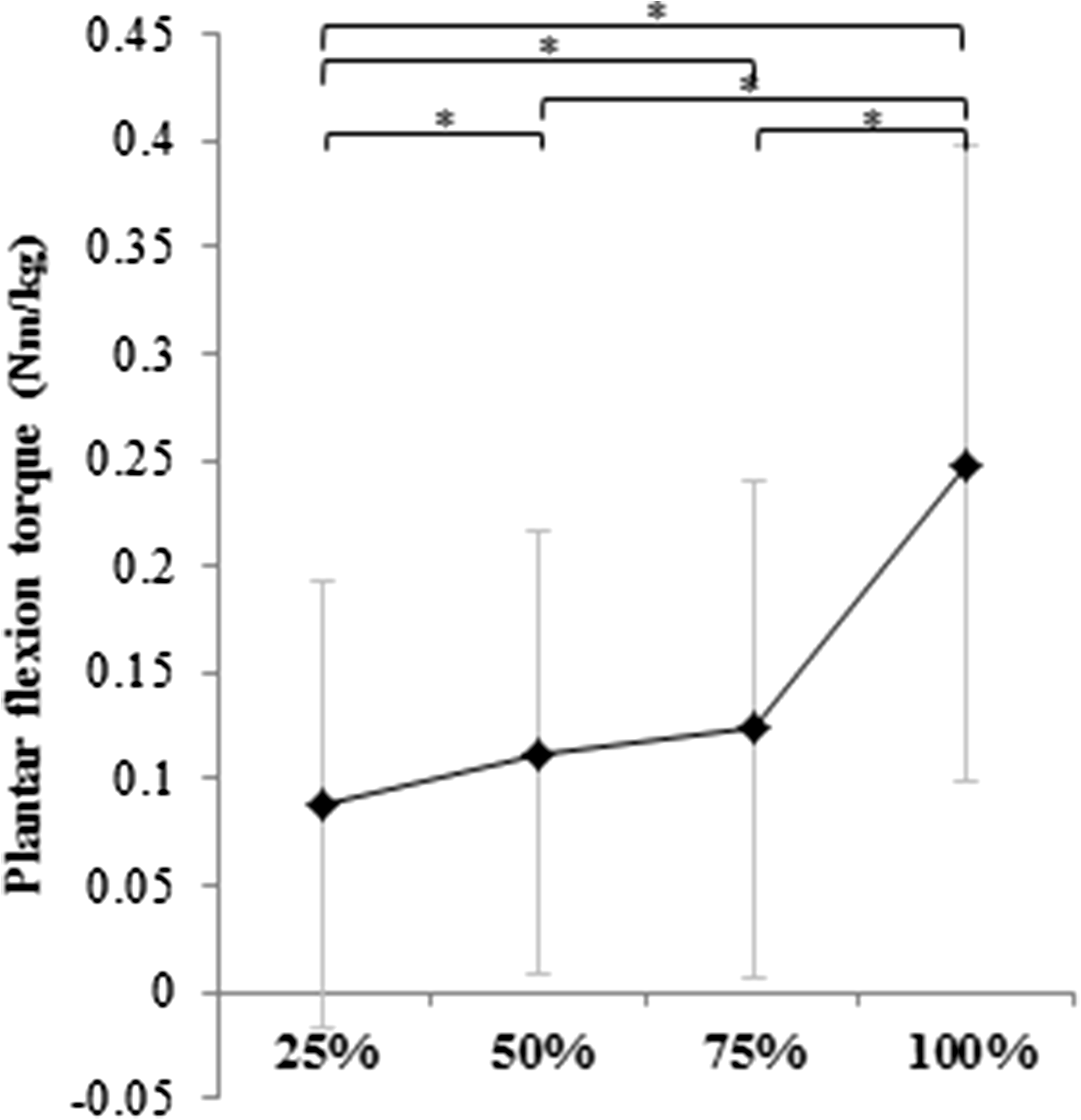

Supplement: Supplementary file 2 — Authors’ original file for figure 2 [file 12984_2014_661_MOESM2_ESM.tif]

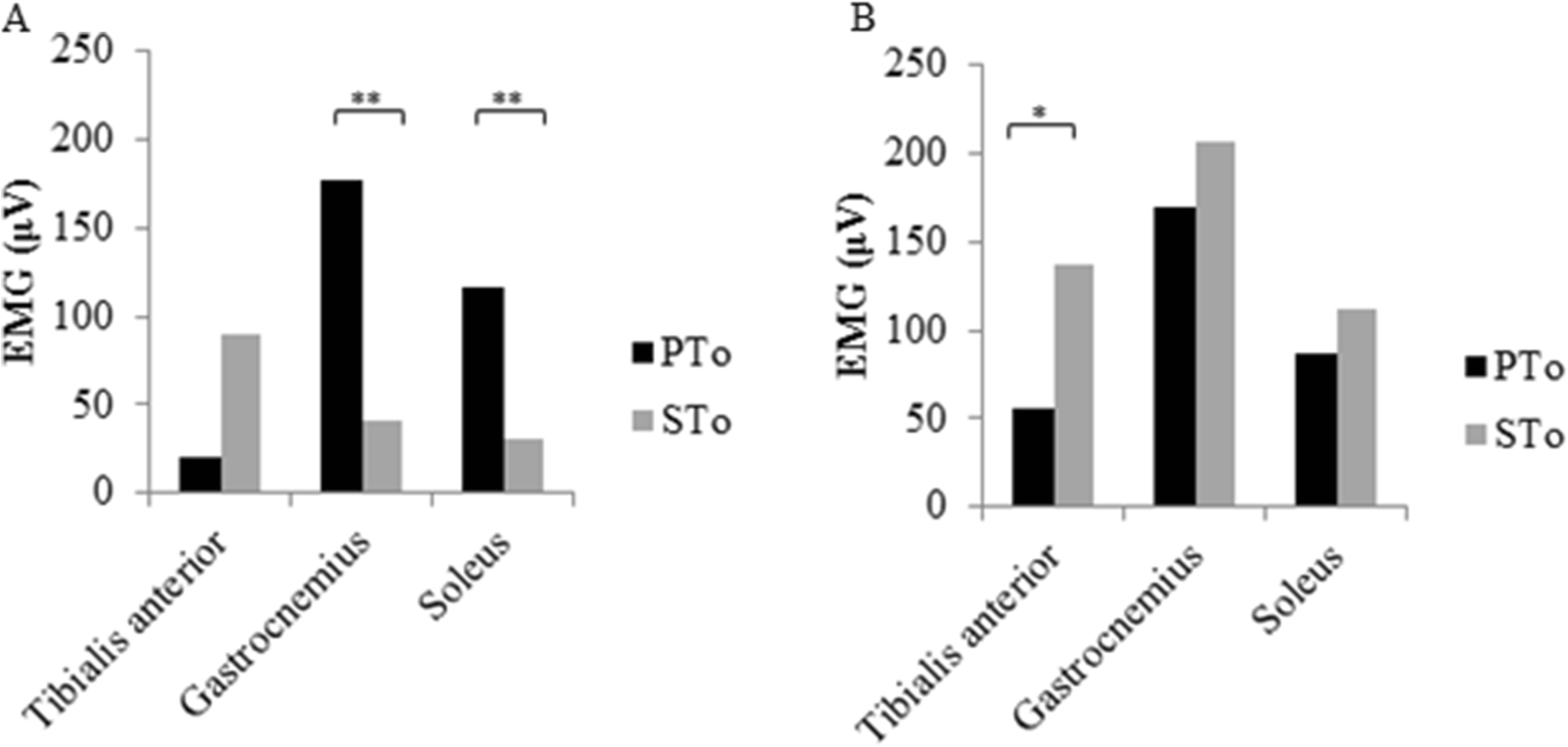

Supplement: Supplementary file 3 — Authors’ original file for figure 3 [file 12984_2014_661_MOESM3_ESM.tif]
